# Enhancing wildlife monitoring with computer vision: A dataset for automated detection of barbary macaques

**DOI:** 10.1016/j.dib.2026.112973

**Published:** 2026-06-13

**Authors:** Kenza Qitout, Clémence Lochin, Xavier Desquesnes, Audrey Maille, Bruno Emile

**Affiliations:** a Laboratoire PRISME, Université d'Orléans, INSA CVL, Orléans 45072, France; b Laboratoire Eco-anthropologie, UMR 7206, MNHN, CNRS, Université Paris Cité, Paris 75016, France

**Keywords:** Artificial intelligence, Deep learning, Multiple object detection, Instance segmentation, Non-human primates

## Abstract

Automated monitoring of animal populations is becoming increasingly important in biodiversity research and wildlife conservation. However, detecting and identifying animals in complex and diverse environments remains a significant challenge. In this study, we introduce *MagotSeg*, a novel dataset designed for the automated detection, segmentation, and classification of Barbary macaques (*Macaca sylvanus*), a north-African primate species classified as Endangered on the IUCN red list, in the loosely environment of a zoo setting. The dataset consists of 1742 annotated images, comprising 6689 individual detections captured from multiple camera angles, at different times of day and across seasons, reflecting a wide range of environmental and lighting conditions. Unlike many existing datasets, *MagotSeg* includes instance-level segmentation and multi-label classification, distinguishing individuals into two age categories. This enables advanced computer vision tasks beyond basic object detection. We used the dataset to train and evaluate the performance of several versions of the YOLO (*You Only Look Once*) object detection model. This resource has potential applications in the automated monitoring of primates and may support conservation-related research through the integration of visual monitoring technologies.

Specifications Table

**Table:** 

Subject	Computer Sciences
Specific subject area	Primate detection and classification in loosely controlled environment
Type of data	Images (.png files), Table (.csv file), Annotations (.txt files)
Data collection	This dataset contains 1742 annotated images with instance segmentation. Image data were collected from raw videos recorded in a zoo enclosure, where cameras were installed for one year: six 1080p sensors with an ultra-wide 194° horizontal field of view and two 4 MP dome cameras equipped with 36× optical zoom and optical image stabilization. Multiple annotations were performed on the dataset images: bounding boxes for object detection, segmentation mask around each individual for instance segmentation and classification. We used images with these annotations to train and test different object detection and segmentation models.
Data source location	ZooParc de Beauval, France 47°14′58.158″N 1°21′10.506″E
Data accessibility	Repository name: data.InDoRES Data identification number: https://doi.org/10.48579/PRO/K7FEET Direct URL to data: https://data.indores.fr/dataset.xhtml?persistentId=doi:10.48579/PRO/K7FEET
Related research article	None.

## 1 Value of the Data

• Advances in camera and video technology are generating large quantities of visual data in natural environments, either passively (camera trapping and video surveillance) or actively (manual recording of video footage). However, extracting behavior information still relies on a time-consuming task and error-prone manual annotation. Developing automated analysis method would significantly reduce effort, improve consistency and enable more efficient use of these datasets for behavioral studies.
• Although, several detection datasets exist, they were generally generated in controlled environments (boxes, cages) [[1], [2], [3], [4]], with limited variability (unlike real-time monitoring throughout the day, all year long) [5,6], few individuals per image, and simple conditions (simple camera viewpoint, no multiple perspectives, few situations), making them unsuitable for detection of many animals in uncontrolled environments. Moreover, existing datasets are centered on one or two visually distinct individuals (e.g. different fur colors in laboratory animals [12]), with a camera viewpoint very close (e.g., MacaquePose [7]), limiting the detection to complex environment with many similar individuals, occlusions and outdoor conditions.
• Existing datasets are generally designed for a single task as detection or pose estimation. For example, AP-10 K [8] contains around 10 K images across 54 species with pose annotations, while MacaquePose [7] includes 13 K images focused on primates with pose and segmentation annotations, but not detection, and with up to three individuals per image. Other datasets provide larger collections, such as up to 180 K images for detection (e.g., Pan50K [9]), or >30 K videos and 33 K images for pose estimation across hundreds of species (e.g., Animal Kingdom [10]), but with task-specific or incomplete annotations, a limited number of individuals per image, restricting their use for classification and multi-label detection tasks which remain relatively underexplored [7,[11], [12], [13]].
• *MagotSeg* is an innovative dataset focused on a large group of primates (> 30 individuals) in a loosely controlled zoo environment using several remote cameras and viewpoints with various angles, foregrounds and backgrounds. Videos were recorded continuously under diverse real-world conditions (weather conditions, lighting, exposure and contrast). The studied population can be structured into at least two age classes and exhibits rapid movements with varied and complex individual or social behaviors. This dataset is designed to support the development of reliable automated computer vision systems for detecting multiple primates simultaneously and classifying them by age, enabling the development of tools to analyze behaviors with artificial intelligence. Here, we present baseline detection and segmentation experiments to demonstrate the usability of and potential of *MagotSeg* within standard AI pipelines, without focusing on full model optimization.

## 2 Background

There is an urgent need to develop methods to monitor endangered species in their natural range [14,15], including primates as half of the species is threatened with extinction [16]. In the context of biodiversity management, a major challenge is to identify individuals, which implies recognition of the species or class in a population, and also discrimination of individual features. Although there are different methods of tracking animals, such as live observation, GPS collars or microchips, one effective way is to use real-time visual devices. Indeed, remote cameras allow animal monitoring without physical markers on animals, preventing any disruption of their behavior.

Automated tools have been developed to perform animal detection in images, as this task represent a prerequisite for various tasks such as tracking or behavior recognition. Object detection models have been pre-trained on very large databases and their direct use allows to predict individuals on an image very quickly. However, many problems remain for accurate animal detection in loosely controlled environments. Furthermore, existing datasets of images collected in natural environments do not show a diversity of viewpoints. It is thus necessary to fine-tune these models with a database corresponding more to animals evolving in the natural world.

## 3 Data Description

### 3.1 Image retrieval

**Fig. 1:**
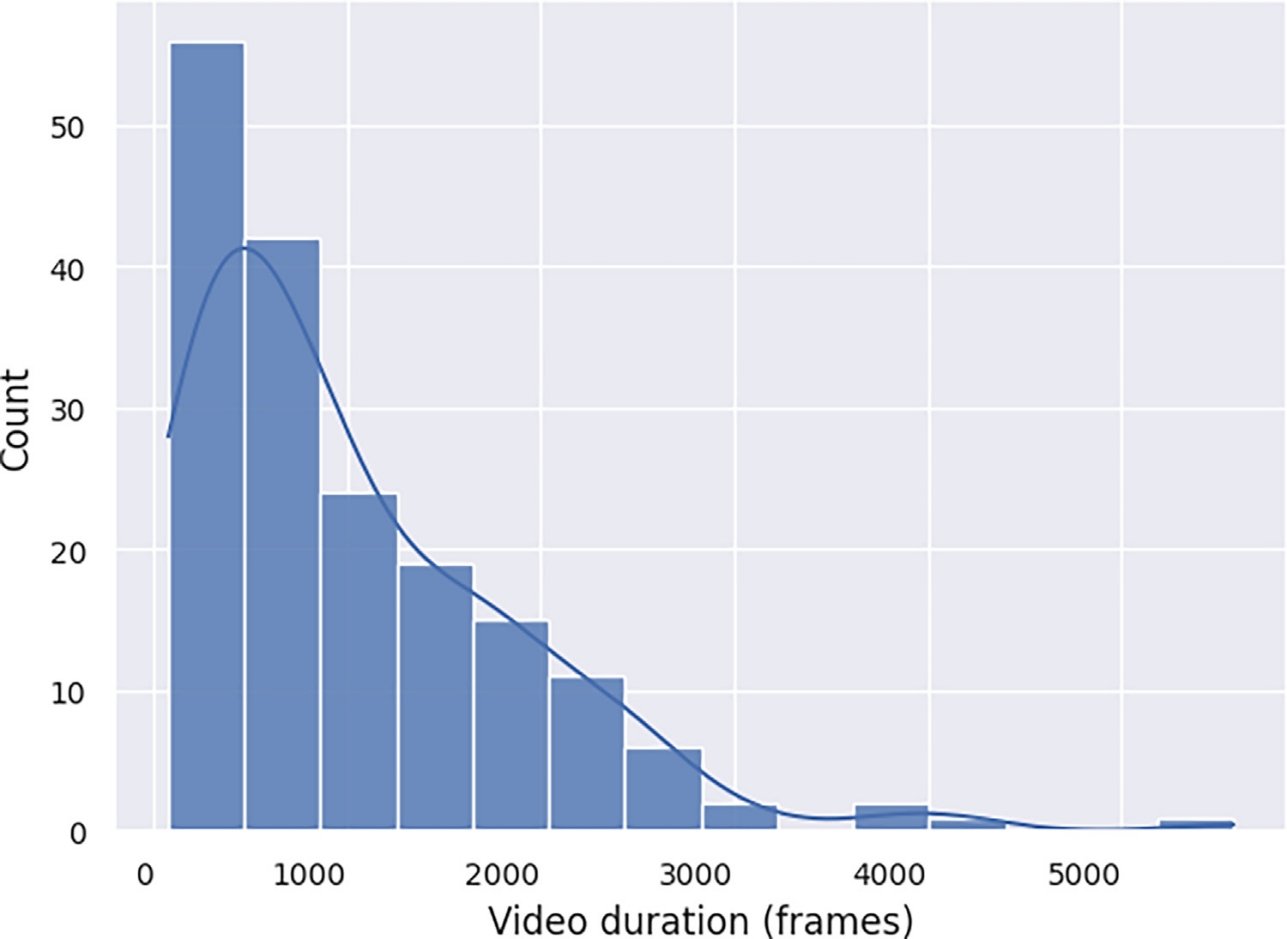
Histogram of distribution of video duration (in frames number). The line corresponds to a kernel density estimate (KDE).

The dataset was built using video sequences from raw videos recorded in a zoo enclosure. A total of 179 videos were extracted from the seven video cameras (Table 1) or eleven video camera angles, with an average of 1003.09 ± 685.8 images in videos. There are no notable differences between the distribution by video camera and by video camera angle (Fig. 1).

**Table 1:** Table of video number per video camera.

Video Camera	Video	Images	Type	Angle
Video Camera 1	20%	20%	Stationary	wide shot
Video Camera 2	26%	27%	Stationary	wide shot
Video Camera 3	17%	18%	Stationary	wide shot
Video Camera 4	19%	17%	Stationary	wide shot
Video Camera 5	2%	1%	Stationary	wide shot
Video Camera Dome	16%	17%	Mobile	close-up

After collecting videos for each video camera, the next step was the extraction and selection of images. As the objective was to build a dataset of images that could be used to train a model for individual detection, it was important to identify images of interest from the videos, which were images containing Barbary macaques. Then, to avoid overfitting from images derived from a small number of videos, which were more prone to be similar, the image selection was conducted manually, starting from the first image with initial situation. Each image in the video presenting a new situation was selected; for example, when an individual moves in the video, when one individual is missing or a new individual appears in the image or when an individual is in a new position. This process continued until the last image of the video was reached, ensuring that all images selected from a video were different from one another. In order to preserve the raw characteristics of the data, all images were kept in their original resolution as acquired (this approach allows users to apply their own preprocessing strategies depending on the requirements of their models and applications).

Additionally, data on the day and time of the video was recorded, and the image number within the video was retained.

As considering age classes of animals provides valuable information about the studied population for biological sciences, particularly ecology, we aimed to classify the Barbary macaques into two categories: *mature* and *juvenile* individuals corresponding to the population structure based on age classes. However, as few juveniles were present in the group, there was an under-representation of the *juvenile* class in the dataset. To avoid class imbalance, videos (and their corresponding images) featuring multiple juveniles were selected to increase the detection number in the *juvenile* class.

**Fig. 2:**
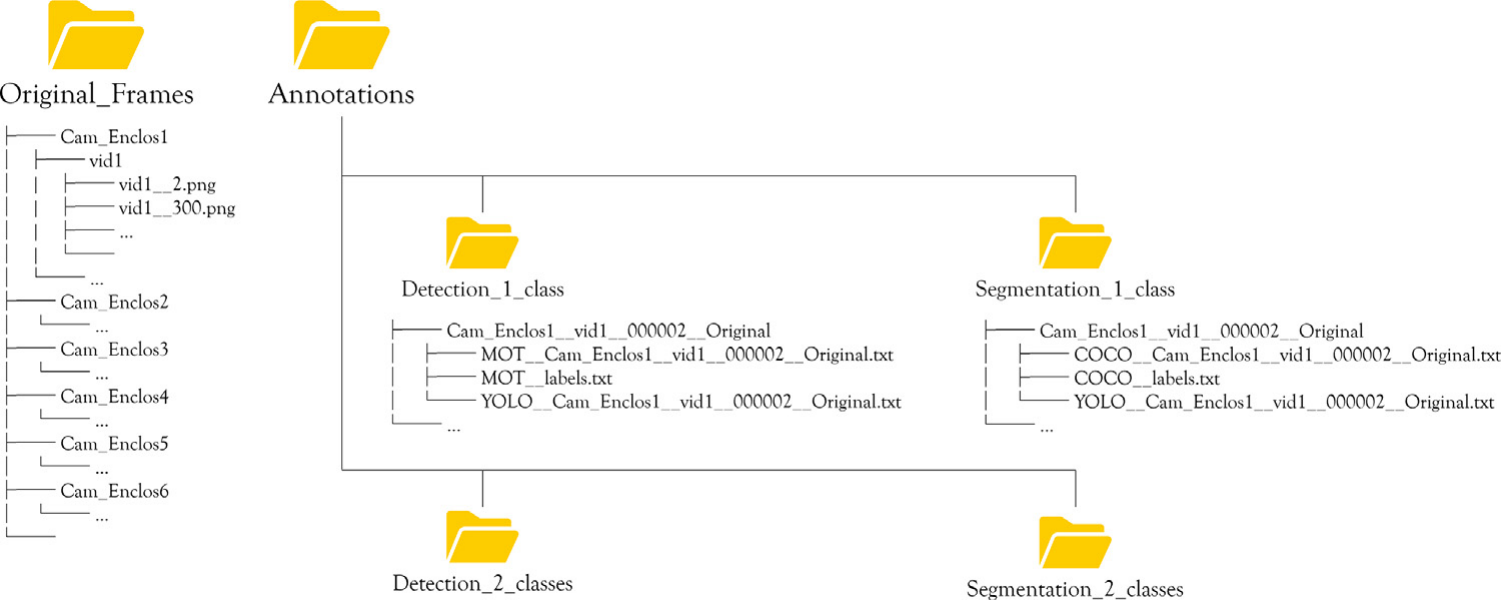
Dataset organization.

The dataset is organized into two subdirectories with a CSV file (Fig. 2).

− The images extracted from videos are stored in the *Original_Frames* subdirectory, which is organized by camera and by video, and are provided as raw, unprocessed images. All images are PNG files and share the same filename as the video they originate from, with the frame number appended, following the format *vlc-record-2023–11–17–15h49m30s-d03_20,231,115,114,752__2.png*. Filenames include information about the camera, the date and time of acquisition, and the date and time when the video was stored on the server.
− The annotations of images are stored in the *Annotations* subdirectory, which is organized into four subfolders: *Detection_1_class, Detection_2_classes, Segmentation_1_class*, and *Segmentation_2_classes*. The *Detection_2_classes* and *Segmentation_2_classes* subdirectories contain the same files as *Detection_1_class* and *Segmentation_1_class*, respectively, but considering the binary classification between *mature* and *juvenile* individuals. Annotation files are named according to the annotated image filename, including a suffix indicating the data type (here *Original*), which refers to the unmodified images. Annotation (TXT files) are available in COCO, MOT, and YOLO formats, with class labels specified in the *MOT_labels.txt* and *COCO_labels.txt* files. The COCO-format annotations correspond to coordinates of segmentation polygons that outline individuals. The COCO-format annotations correspond to coordinates of bounding boxes of individuals. The YOLO-format annotations correspond to coordinates of segmentation polygons and bounding boxes in the YOLO model format.
− The CSV file *videos_infos.csv* contains summary of weather conditions for each video

### 3.2 Statistics

**Fig. 3:**
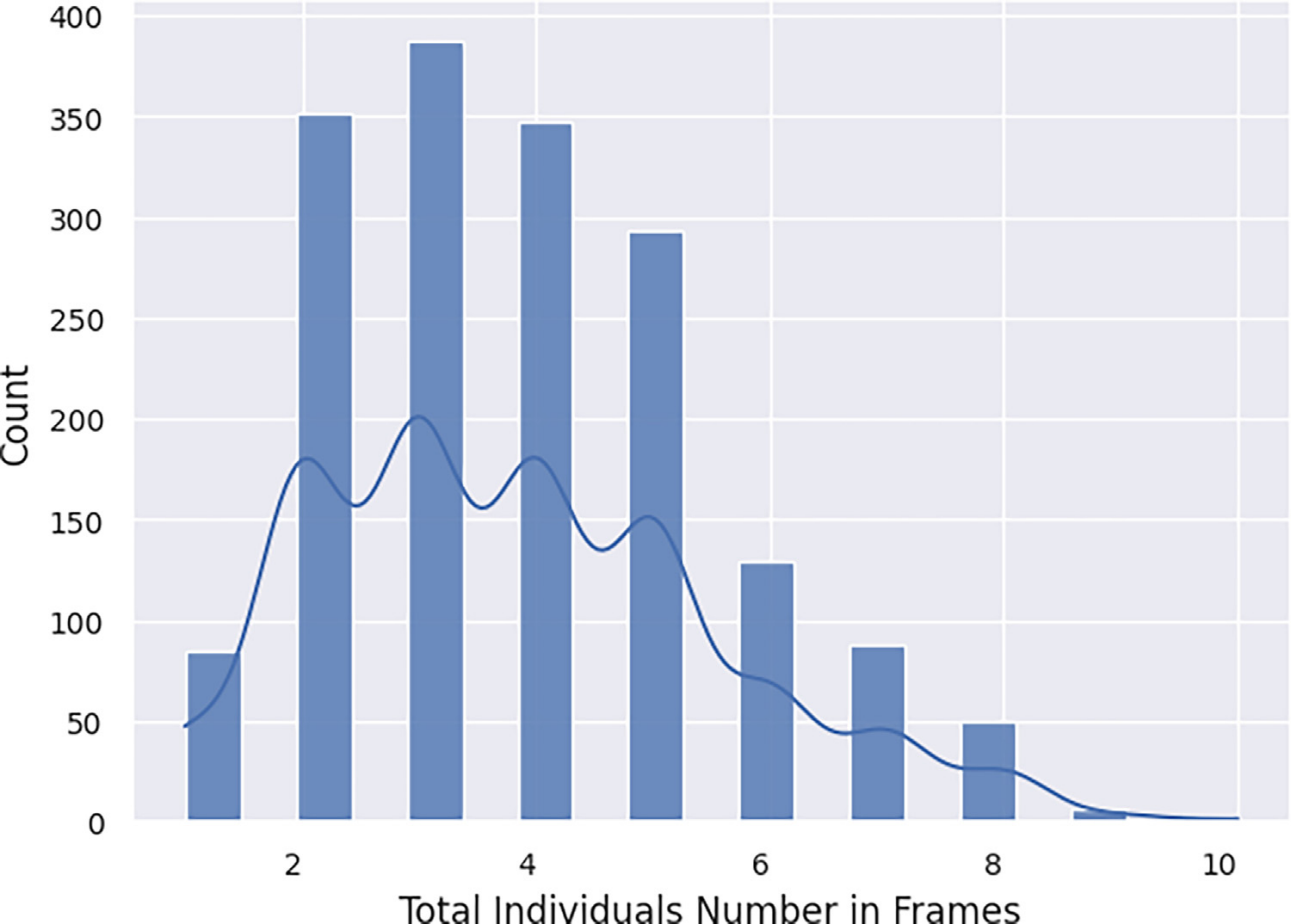
Histogram of distribution of the total number of individuals per frame. The line corresponds to a kernel density estimate (KDE).

**Fig. 4:**
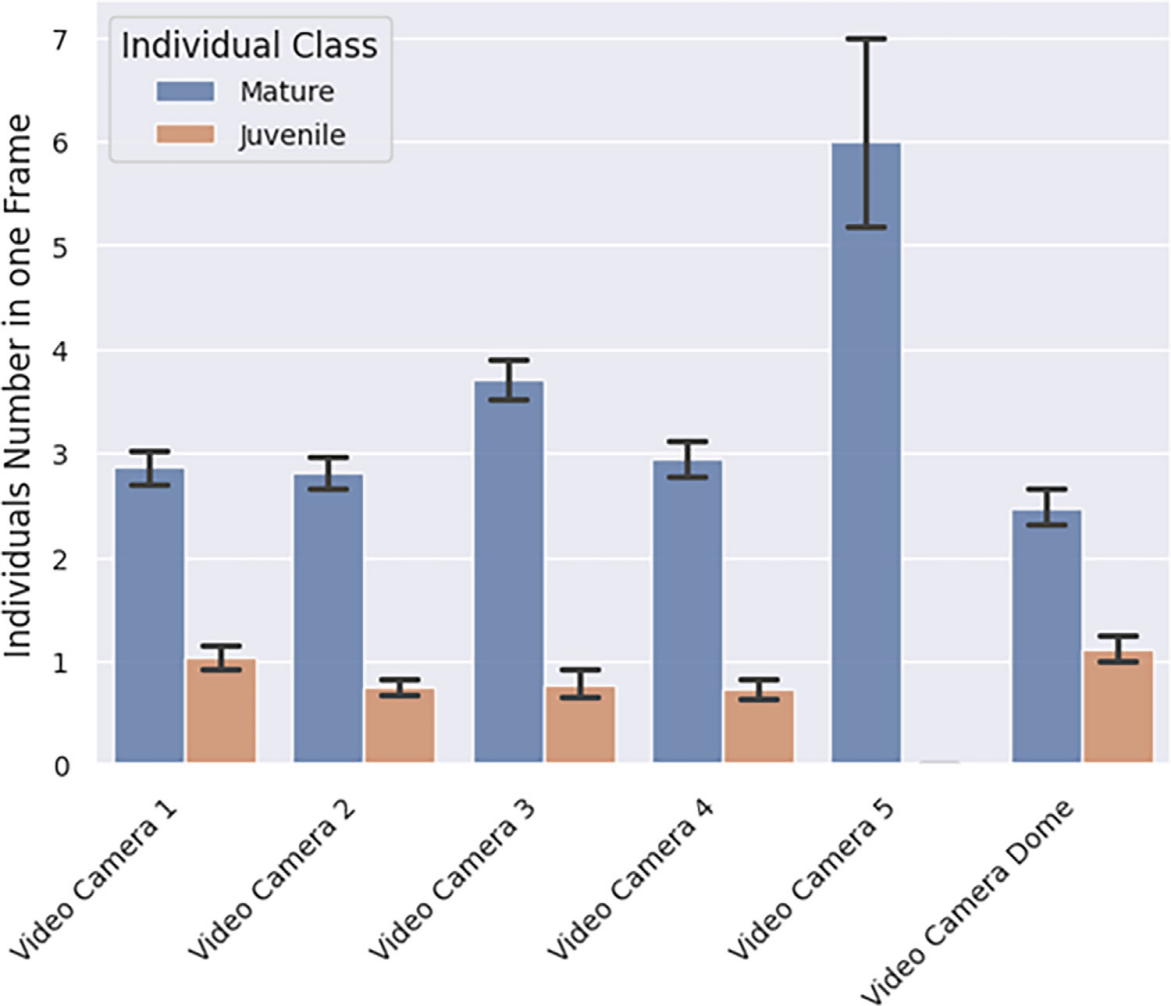
Histogram of the number of individuals per frame according to individual classes (*mature* or *juvenile*) for each camera.

The final dataset contains 123 min of video and 1742 annotated images from 179 videos. The dataset includes 6689 segmentation polygons, with 5183 polygons for the *mature* class and 1506 polygons for the *juvenile* class. On average, the images contain 3.8 individuals (Fig. 3) with class imbalance between *mature* and *juvenile* individuals (Fig. 4). The average duration of original videos is 41 s with a FPS of 24. The dimensions are mostly the same for all images, namely 1920×1080.

Different weather conditions and time of the day between all images create shadows and contrast, as well as differences between the foreground and background, and varying exposure. The vast majority of images shows a basic weather without any particular climatic event, but there is a diversity of possible combinations of climatic conditions with 846 images showing a dull appearance and 896 images having a bright or colorful appearance. The sun is present in 504 images and it rains in 84 frames. The distribution of seasons across all video cameras shows a significant imbalance, with a large majority of frames captured during winter across all cameras combined. Images from videos taken in summer and spring have a different saturation with a very colorful appearance. The distribution of the time of day when frames are captured is balanced across all video cameras, although the video camera 3 has relatively few images taken in the morning. Table 2 summarizes frequencies of different weather conditions and contexts present on all the images in the dataset.

**Table 2:** Table of different weather conditions and contexts of frames.

Context	Value	Frequency
Weather Conditions	Sun Shadows Rain Blur Backlight	504 349 84 70 90
Lighting	Dull Colorful Bright	846 646 250
Time Day	Morning Noon Afternoon	491 552 699
Season	Winter Spring Fall	990 553 151
Resolution	1920×1080 2560×1440	1678 64

The dataset displays the initial images providing coordinates of segmentation polygons and bounding boxes, and class labels. 889 images contain at least 1 bounding box for *juvenile* while 853 images contain only bounding boxes for *mature* individuals. 85 images contain a single individual (with 51 are *mature* individuals and 34 are *juvenile* individuals), while 1380 images contain between two and five individuals, and 570 images contain more than five individuals with three images that contain the maximum of 10 individuals.

### 3.3 Object detection results

The dataset was tested to train the YOLO models, namely *yolov3, yolov5* and *yolov8* versions for object detection.

We have fine-tuned these 3 models for detection and classification of Barbary macaques in 2 age classes. We evaluate models with the mean Average Precision (mAP). The results are on average 0.52 with a maximum of 0.77 for *yolov5*, a learning rate of 0.001 and a batch size of 8. Differences are observed with the average mAP for the video camera 3 and mobile video camera, the lighting bright, the time day morning, weather conditions shadows, backlight and rain which have an average mAP at least 10% lower than the overall average mAP. Results show a difference between the two classes with *mature* individuals better detected than *juvenile* individuals, potentially due to the class imbalance that exists in the database. By analyzing prediction errors, we observed that *juvenile* individuals could be detected but were misclassified. Indeed, the prediction of *mature* class shows better performance for all trained models with an average AP of 0.67. Conversely, the prediction of *juvenile* individuals shows an average AP of 0.37.

To reduce this class imbalance, data augmentation was applied to images with at least one *mature* individual. We fine-tuned the 3 models on the same distribution of the 5 data folds (Table 3). The results do not show significant differences and remain low compared to previous results and the results presented in the literature.

**Table 3:** Table of mean mAP scores for object detection and instance segmentation models trained on two classes with data augmentation.

Model	Batch size	Learning rate	Confidence score	Object detection (mAP)	Instance segmentation (mAP)
YOLOv3	8	0.01	0.5	0.67	-
YOLOv3	8	0.001	0.7	0.64	-
YOLOv5	8	0.01	0.5	0.65	0.40
YOLOv5	8	0.01	0.7	-	0.19
YOLOv5	8	0.001	0.5	-	0.21
YOLOv5	8	0.001	0.7	0.62	0.04
YOLOv8	8	0.01	0.5	0.64	0.38
YOLOv8	8	0.01	0.7	-	0.18
YOLOv8	8	0.001	0.5	-	0.38
YOLOv8	8	0.001	0.7	0.59	0.24

**Fig. 5:**
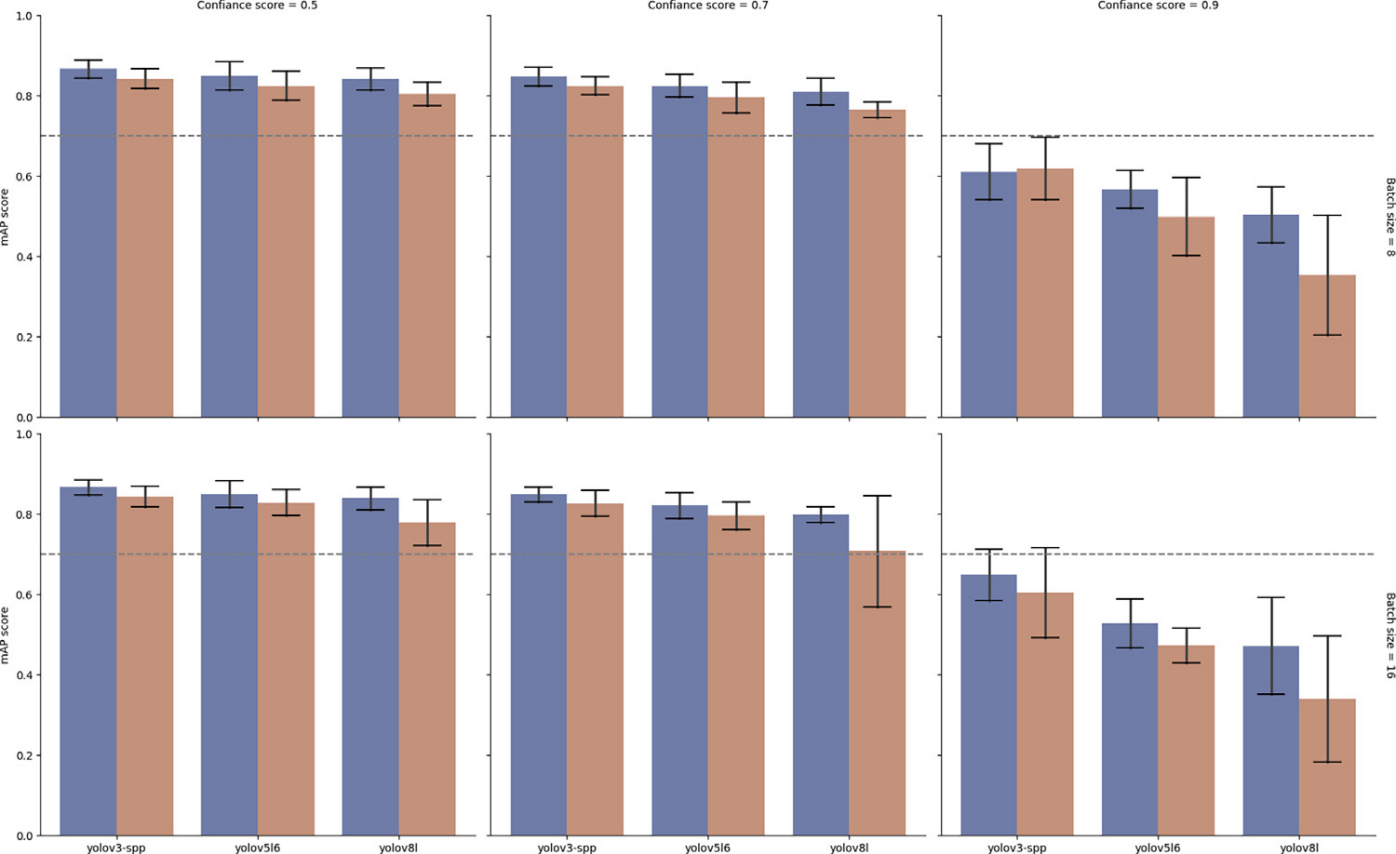
Histogram of mean mAP scores for object detection models trained on one class. Blue: *lr*=0.001; Orange: *lr*=0.01.

To no longer depend on classification errors and only test the individual detection, we fine-tuned the 3 models without considering the classes. Performances are better with an average mAP of 0.72 with a maximum at 0.90 for YOLOv3, a learning rate of 0.001 and a batch size of 8 (Fig. 5).

There is no observed difference with the average mAP depending on the video camera, lighting, time the video camera, lighting, time day, season, weather conditions except for camera video 5 and the weather condition backlight which have an average mAP 10% lower than the overall average.

### 3.4 Instance segmentation results

**Fig. 6:**
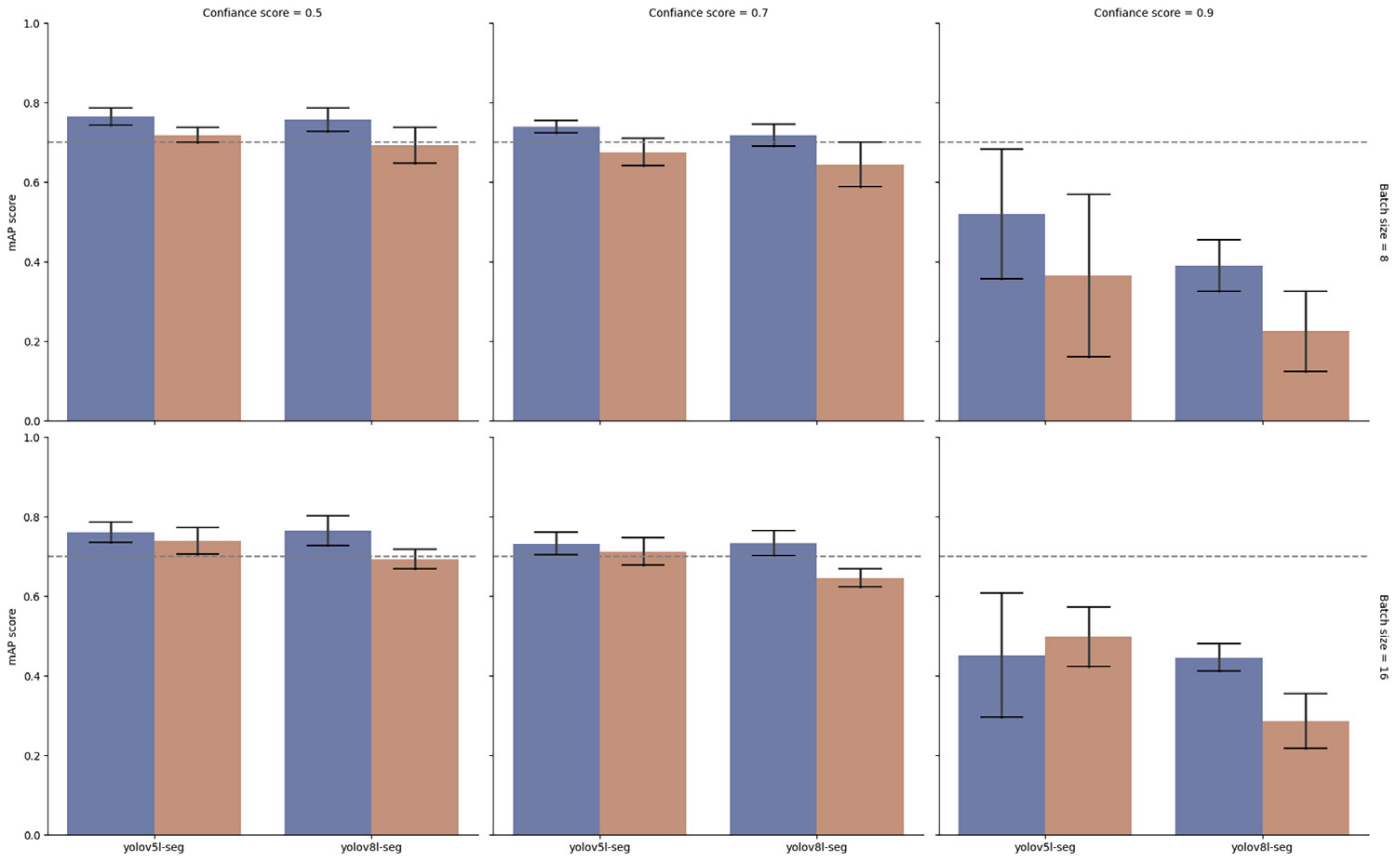
Histogram of mean mAP scores for instance segmentation models trained on one class. Blue: *lr*=0.001; Orange: *lr*=0.01.

The dataset was tested to train the YOLO models, namely *yolov5* and *yolov8* versions for instance segmentation. We have fine-tuned these 2 models for segmentation and classification, with data augmentation on *juvenile* individuals (Table 3), or no classification (Fig. 6).

For segmentation on 2 individual classes, the results are on average 0.44 with a maximum of 0.71 for *yolov8*, a learning rate of 0.001 and a batch size of 8. Differences are observed with the average mAP for the video camera 2, video camera 3 and video camera 5, the lighting conditions, the season fall, weather conditions backlight, rain, shadows and blur which have an average mAP at least 10% lower than the overall average mAP. Results show a difference between the two classes with *mature* individuals better detected than *juvenile* individuals with an average AP of 0.58 for the prediction of *mature* and an average AP of 0.30 for the prediction of *juvenile*.

The results of data augmentation do not show significant differences and remain low compared to previous results after we fine-tuned the 2 models on the same distribution of the 5 data folds (Table 3).

For segmentation without classification, we fine-tuned the 2 models without considering the two age classes. Performances obtained are better with an average mAP of 0.61 with a maximum at 0.80 for YOLOv8, a learning rate of 0.001 and a batch size of 16 (Fig. 6).

There is no observed difference with the average mAP depending on the video camera, lighting, time the video camera, lighting, time day, season, weather conditions.

## 4 Experimental Design, Materials and Methods

### 4.1 Biology model and study site

The animal species studied here is the Barbary macaque, a primate of the *Cercopithecidae* family. The study site is ZooParc de Beauval, with a focus on the Barbary macaques' enclosure covering an area of approximately 600 m² and equipped with big rocks and wooden structures. At the time of the data collection, the enclosure contained between 33 and 37 individuals, including 3 to 5 juvenile individuals. Three age classes are distinct yet very similar in this species of primates: large adult individuals that have reached full body size (between 45 cm and 60 cm) with light brown fur, large sub-adult individuals with a morphology comparable to that of adults but that did not reach sexual maturity and small juvenile individuals that are smaller with long upper limbs and fur color ranging from dark gray to light browndark .

As sub-adults and adults are very difficult to differentiate in videos, dataset contains information on two classes only, mature individuals (for adult and subadults) and juvenile individuals, hereinafter designated as matures and juveniles.

**Fig. 7:**
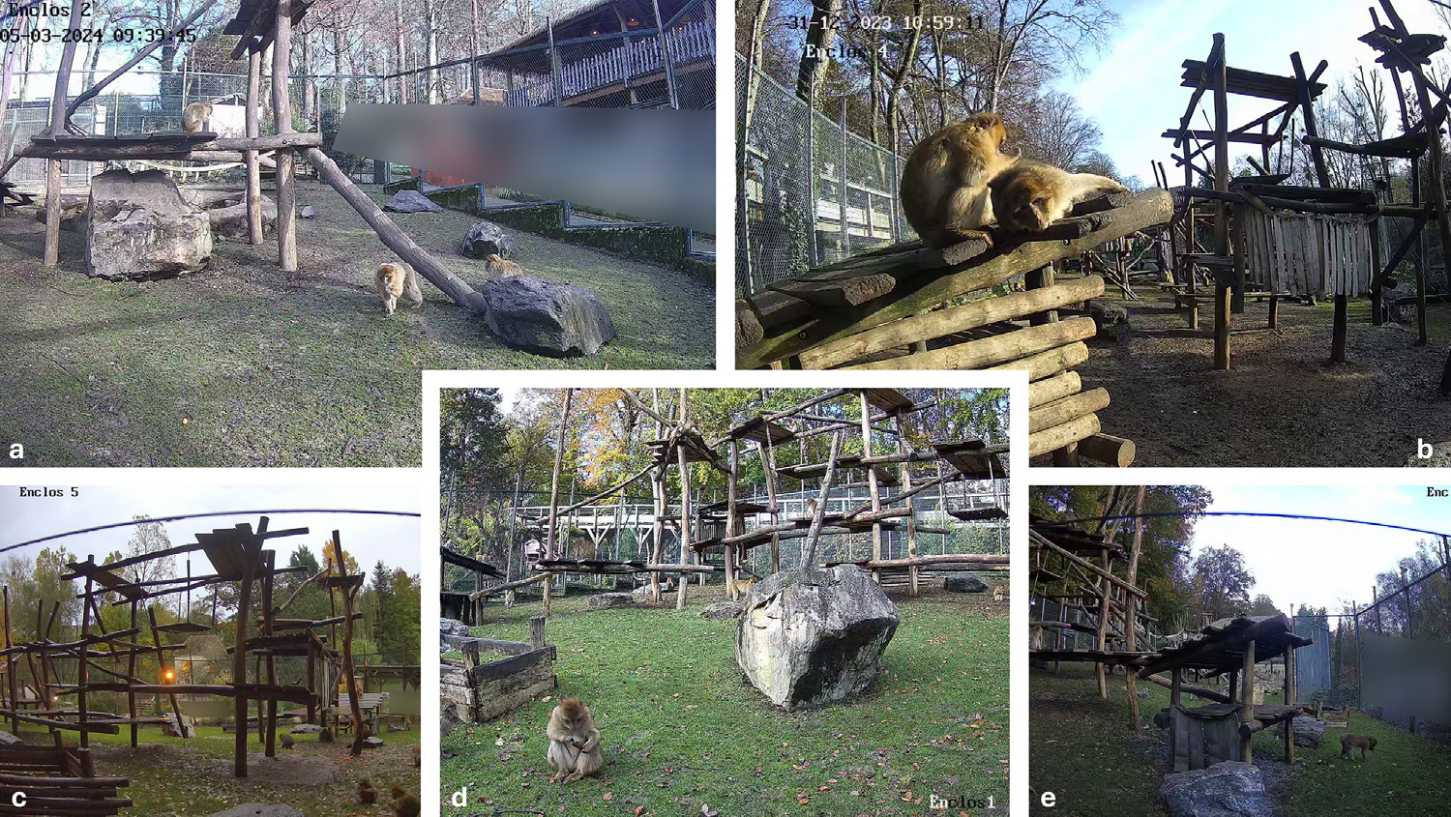
Raw images extracted from videos recorded by each of the five stationary video cameras under different conditions as defined in Table 2. a) Camera 2, *Dull* and *Shadows*; b) Camera 4, *Bright, Sun* and *Shadows*; c) Camera 5, *Rain* and *Blur*; d) Camera 1, *Colorful* and *Sun*; e) Camera 3, *Dull* and *Backlight.*

**Fig. 8:**
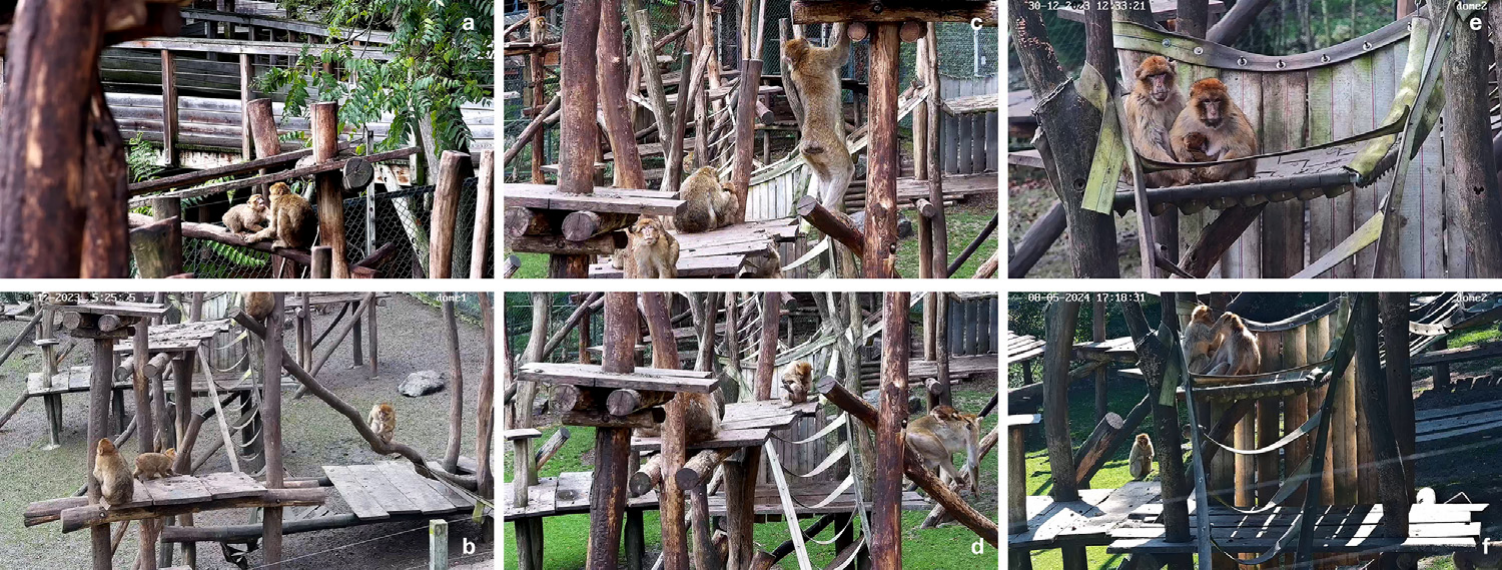
Raw images extracted from videos recorded by two mobile video cameras, each with three different angles and a gradient in zoom levels, under different conditions as defined in Table 2. a) *Colorful*; b) *Dull*; c) *Colorful*; d) *Colorful*; e) *Dull*; f) *Colorful, Sun, Shadows* and *Backlight.*

The enclosure is monitored with five stationary video cameras and two mobile video cameras, providing a diversity of viewpoints that are captured in the dataset. The viewpoint of our video cameras features more or less wide angles, which results in individuals often being partially (or completely) hidden by obstacles. The five video cameras are wide shots (Fig. 7) and the two mobile cameras present close-ups with a total of six different angles (Fig. 8). Given the diversity of angles of mobile video cameras, all the images from these cameras have been grouped into a single group.

This acquisition setup was chosen to increase the diversity of the dataset by capturing both close-up and distant observations, thereby including images of varying quality, from sharp details to more challenging blurred conditions. Both mobile and fixed cameras were used, enabling the acquisition of images from diverse viewpoints and introducing natural variations in angles, positions, and backgrounds across the dataset. Despite this variability, the images from mobile cameras consistently focus on the individuals, providing high-quality visual information with detailed features.

These video cameras provide real-time video streams to allow monitoring of multiple individuals simultaneously. Videos are recorded at different times of the day, then saved and stored on a server.

### 4.2 Annotations

**Fig. 9:**
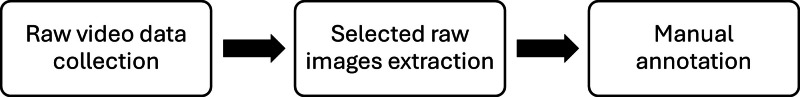
Overview of the process in three steps: (1) collection of raw video data, (2) randomized selection of images and extraction of the selected raw images, and (3) manual annotation of images.

All frames were initially extracted from the videos. A manual selection was then performed to ensure the quality and diversity of the dataset (Fig. 9). This selection included both sharp and moderately blurred images to reflect realistic acquisition conditions. However, images that were excessively blurred or affected by strong backlighting from camera 5 were excluded, as they did not allow for a clear visual distinction of the individuals.

**Fig. 10:**
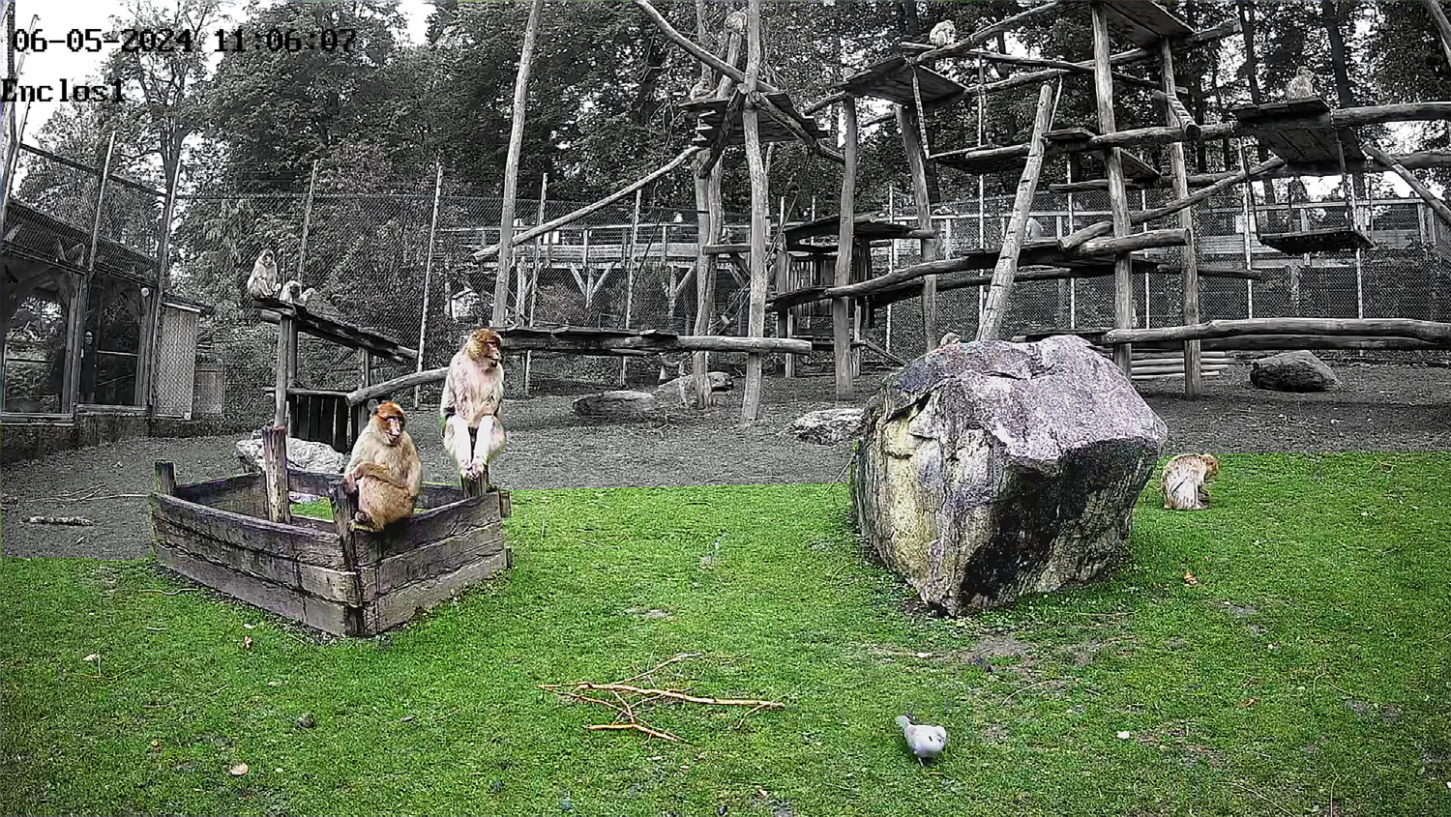
Raw image with manual delimitation of the annotation area restricted to the foreground.

**Fig. 11:**
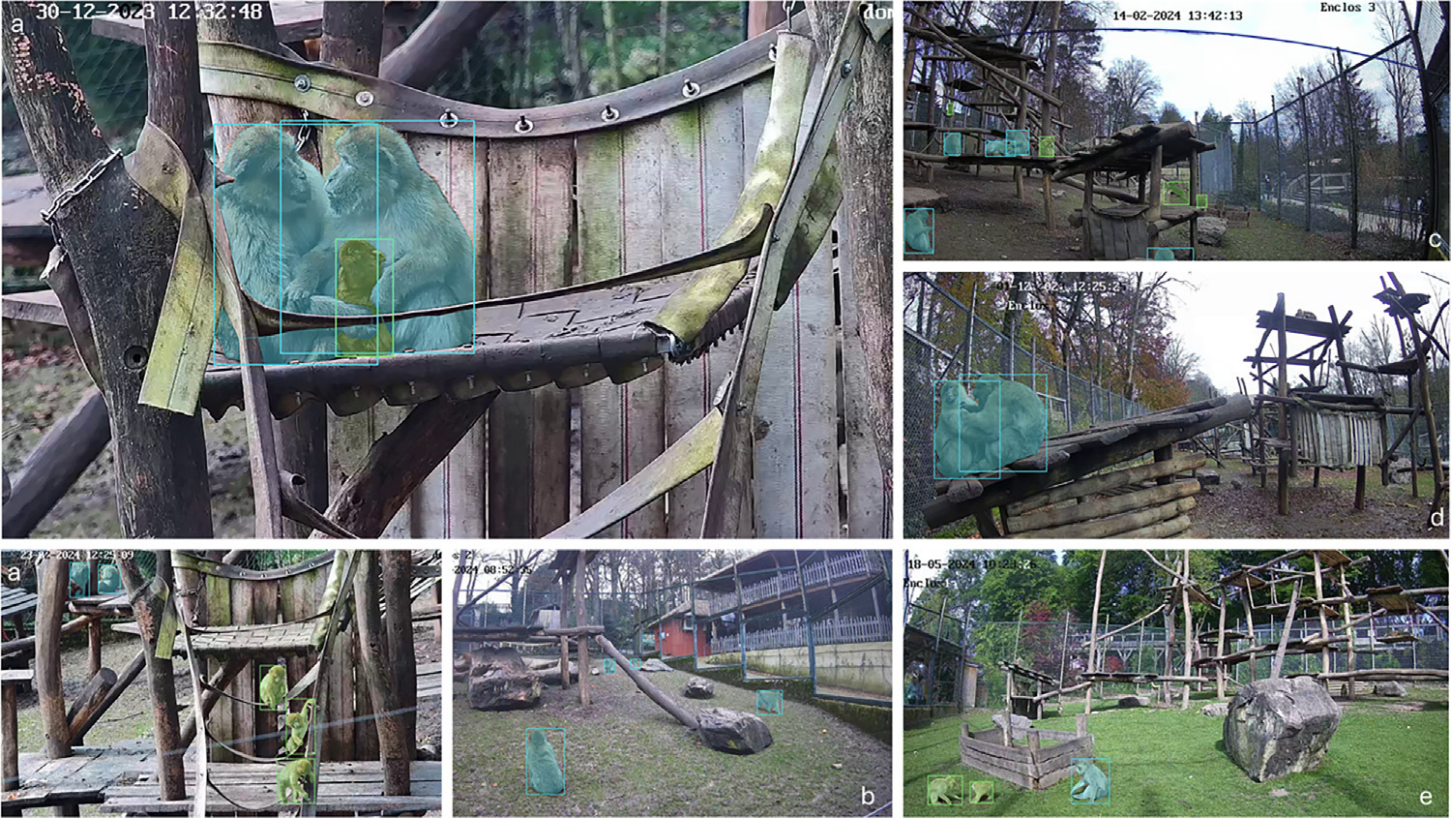
Annotations of bounding boxes and segmentation masks of individuals on the two classes. Blue: *mature*; Green: *juvenile.*

**Fig. 12:**
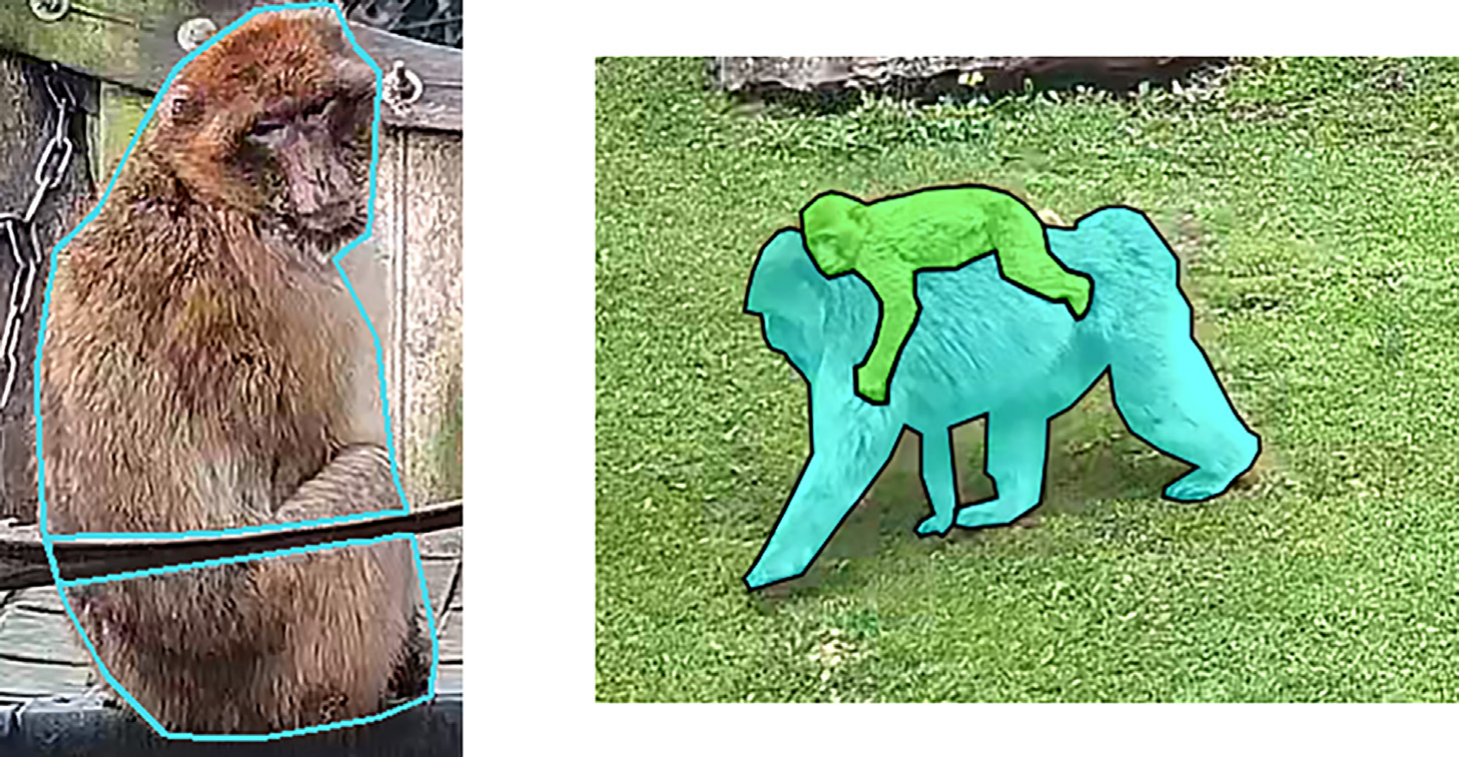
Rules for segmenting individuals. Blue: *mature*; Green: *juvenile.*

On each selected image, annotations were performed for all individuals. It was decided that only the individuals in the foreground would be annotated in order to provide accurate annotations, particularly for individual segmentation. Indeed, the image quality does not allow to accurately outline the contours of individuals in the background because they are too small, hidden and barely visible, even to the naked eye, to see their different body parts (fingers, limbs, arms) and body actions (for example, facial expressions). Keeping only the foreground allows individuals to appear larger and closer to the video camera. Therefore, we defined an annotation zone in the foreground for each video camera (Fig. 10). Annotations include individual segmentation masks defined as contours of each individual (Fig. 11). In cases where an obstacle was in front of an individual, the two parts of the individual were connected by a line. In cases where two individuals were in contact, contours have been made so that all the points in contact touch each other (Fig. 12). From segmentation masks, bounding boxes were calculated as the boxes containing all the segmentation points.

Only a small number of images from the video camera 5 (Table 1) were annotated due to poor image quality resulting from backlighting and preventing precise segmentation. However, to retain an overview of all possible situations from all viewpoints, 11 images with 66 annotations from this video camera were used which allows the inclusion of low-quality images and increases model's generalization capabilities.

Manual annotations were performed on the CVAT software with polygon shapes by five annotators, including one expert in Primatology (CL) and one expert in ecology and computer vision (KQ) who checked all the annotations. A second verification of the age-sex class annotation was performed on the entire dataset by another expert (AM).

### 4.3 Image base

All annotated frames were retrieved from the video cameras, resulting in 1742 frames containing 6689 segmentation masks. Three tasks can be performed with this dataset:

1. Individuals detection in images from the videos
2. Individuals segmentation in images from the videos
3. Individuals classification into 2 age classes

The test models panel for each experiment followed the same protocol:

The dataset was divided into training, validation, and test sets, with the test set kept completely unseen for final evaluation. To ensure robustness and reduce bias, we applied a stratification on the data based on the camera viewpoint. Then, the dataset was first partitioned into 10 equal folds, which were then combined differently to create training (70%), validation (10%), and test (20%) sets for each experiment. This process was repeated across 5 independent splits, allowing all samples to participate in different roles while maintaining a rigorous and unbiased evaluation. It is important to note that the frames were selected to ensure diversity. Indeed, they are not necessarily independent, particularly frames from the same video present the same context even if individuals in the frames are in different positions and locations. To avoid having similar frames from the same video in training and test datasets, the three datasets separation was not conducted randomly but in such a way that frames from the same video did not appear in another dataset. This precaution effectively ensures that there are unseen data during training in the test dataset.

We fine-tuned models by varying hyperparameters like the learning rate (0.01, 0.001), the batch size (8, 16) and the optimizer (SGD). Hyperparameters were adjusted according to the validation dataset. The training was stopped when the loss stabilized (Early Stopping) by taking a high number of epochs of 1000 to reach the model convergence.

To increase the data amount, data augmentation techniques were tested on all frames for experiments on one class and on frames with at least one *juvenile* for experiments on two classes. Another dataset of 15,998 frames was created from the database presented in Fig. 2, with filenames indicating the data augmentation technique applied (*Original*: no data augmentation, *HorozontalFlip, RotationFlip, Crop, Blur, Gray, Brightness, Exposure, Contraste, Saturation, Noise*). Each original image was subjected to these geometric transformations. The corresponding annotation coordinates were adjusted accordingly to reflect these transformations. This process generated new images along with updated annotations, in addition to the original images, increasing the size and diversity of the dataset.

The experiments were conducted in a virtual environment under Python 3.11.9, with two GPU NVIDIA TITAN RTX with 24 GB and the library torch 2.2.1. Models were implemented with pip 24.0 and the model code is available in cited articles.

### 4.4 Evaluation metrics

Predictions were evaluated with a confidence score of 0.5. According to articles, the mean Average Precision (mAP metric) [17,18] was used to evaluate models after converting predictions to a standardized format. The mAP metric measures were calculated according to the threshold distance of the bounding box. Here, it was fixed at the threshold to alpha = 0.5, so mAP^0.5^. The mAP metric is calculated with the formula:

(1) $$mAP=\frac{1}{N}\sum_{k=1}^{n}AP_{k}$$

where *AP_k_* is the AP of class *k* and *n* is the number of classes with the AP metric formula:

(2) $$AP=\sum_{n}\left[Recalls\left(n+1\right)-Recalls\left(n\right)\right]*Precisions\left(n+1\right)Recalls\left(n=0\right)=0,\;Precisions\left(n=0\right)=1$$

where *n* is the index of the precision–recall curve.

Each training was performed on all five train, validation and test dataset distributions. The average mAP metric of each training is calculated with the 95% confidence interval.

### 4.5 Object detection

We selected the most performant object detection model according to the state of the art, YOLO [19] because it is faster than existing alternatives

Results obtained were compared for the models *yolov3-spp, yolov5l6* and *yolov8l*. The training was initialized using weights from pre-trained models on the COCO dataset. It is more interesting to use pre-trained models than models initialized randomly. The object detection focused on a single class to test only the individual detection. The predictions of trained models were evaluated on the entire test dataset, depending on each video camera and each climatic condition.

### 4.6 Instance segmentation

The training used the same models panel as the first object detection protocol with the identical data distribution across the five splits on one age class.

Results of mAP^0.5^ obtained for the models *yolov5l-seg* and *yolov8l-seg* were compared. The training was with initialized using weights from pre-trained models on the COCO dataset.

### 4.7 Classification

The training used the same models panel as the first object detection protocol with the identical data distribution across the five splits on two individuals classes.

Results of mAP^0.5^ obtained were compared across all classes, as well as for each individual class and without considering the class (e.g., by labeling all individuals as *mature*).

## Limitations

Our dataset is relatively small compared to large animal behavior datasets [8,10,20]. Furthermore, it may lack variations in environmental conditions because we mostly focused on the Winter season; this issue could potentially be resolved by taking more diverse images, including data from different seasons. Image quality also constitutes a limitation with some images that underwent encoding, which resulted in a loss of fine details and made accurate annotation more challenging. This degradation may reduce the labeling precision, particularly for small regions or fine boundaries.

The dataset exhibits class imbalance where *juvenile* individuals are underrepresented compared to *mature* individuals, which may affect the statistical representativeness and limit the ability to capture the full diversity of scenarios. This uneven distribution could affect the robustness and generalizability of any analysis performed on the dataset. To address class imbalance, particularly for the underrepresented juvenile class, appropriate machine learning strategies can be applied.

Finally, although *MagotSeg* includes multiple viewpoints, unlike existing datasets, it may not fully reflect the natural environments variability. Images were captured from fixed camera viewpoints in a zoo environment, resulting in similar backgrounds, consistent obstacles, and limited diversity of visual contexts. Consequently, the dataset is less generalizable compared to those from natural environments, where viewpoints, backgrounds, and occlusions vary considerably. To enhance generalization, combining this dataset with other datasets may help increase variability and support the development of more robust models.

## Ethics Statement

The data does not involve human subjects, animal experiments, or data collected from social media platforms. We confirm that we have read and comply with the ethical requirements for publication in Data in Brief. We have blurred certain parts of images with human faces before sharing the dataset publicly, in order to respect General Data Protection Regulation (GDPR) guidelines.

## CRediT Author Statement

**Kenza Qitout**: Conceptualization, Data curation, Investigation, Methodology, Software, Formal analysis, Visualization, Writing-original draft, Writing-review & editing; **Clémence Lochin**.: Data curation, Methodology, Writing-review & editing; **Xavier Desquesnes**: Conceptualization, Methodology, Writing-review & editing; **Audrey Maille**: Conceptualization, Methodology, Writing-review & editing; **Bruno Emile**: Conceptualization, Methodology, Writing-review & editing. All authors reviewed the manuscript.

## Data Availability

data.InDoRESDataset MagotSeg for detection and segmentation of Barbary Macaques in zoos (Original data)
